# Intrapleural Dornase and Tissue Plasminogen Activator in pediatric empyema (DTPA): a study protocol for a randomized controlled trial

**DOI:** 10.1186/s13063-017-2026-0

**Published:** 2017-06-24

**Authors:** Michael H. Livingston, Sanjay Mahant, Felix Ratjen, Bairbre L. Connolly, Kevin Thorpe, Muhammad Mamdani, Ian Maclusky, Sophie Laberge, Lucy Giglia, J. Mark Walton, Connie L. Yang, Ashley Roberts, Anna C. Shawyer, Mary Brindle, Simon J. Parsons, Cristina A. Stoian, Eyal Cohen

**Affiliations:** 1 The Hospital for Sick Children, Department of Pediatrics, University of Toronto, 555 University Avenue, Toronto, ON M5G 1X8 Canada; 2 Dalla Lana School of Public Health, University of Toronto, 155 College Street, Toronto, ON M5T 3M7 Canada; 3 Applied Health Research Centre of the Li Ka Shing Knowledge Institute, St. Michael’s Hospital, University of Toronto, 30 Bond Street, Toronto, ON M5B 1W8 Canada; 4 McMaster Children’s Hospital, McMaster University, 1280 Main Street West, Hamilton, ON L8S 4K1 Canada; 5 Children’s Hospital of Eastern Ontario, University of Ottawa, 401 Smyth Road, Ottawa, ON K1H 5B2 Canada; 6 Centre Hospitalier Universitaire Sainte-Justine, Université de Montréal, 3175 Chemin de la Côte-Sainte-Catherine, Montréal, QC H3T 1C5 Canada; 7 Department of Pediatrics, Division of Respiratory Medicine, British Columbia’s Children’s Hospital, University of British Columbia, 4480 Oak Street, Vancouver, BC V6H 3V4 Canada; 8 Alberta Children’s Hospital, University of Calgary, 2888 Shaganappi Trail NW, Calgary, AB T3B 6A9 Canada

**Keywords:** Empyema, Children, Chest tubes, Fibrinolytic agents, Randomized controlled trial

## Abstract

**Background:**

A randomized controlled trial of adults with empyema recently demonstrated decreased length of stay in hospital in patients treated with intrapleurally administered dornase alfa and fibrinolytics compared to fibrinolytics alone. Whether this treatment strategy is safe and effective in children remains unknown.

**Methods/design:**

This study protocol is for a superiority, placebo-controlled, parallel-design, multicenter randomized controlled trial. The participants are previously well children admitted to a children’s hospital with a diagnosis of empyema requiring chest tube insertion and fibrinolytics administered intrapleurally. Children will be randomized after the treating physician has decided that pleural drainage is required but prior to chest tube insertion. After chest tube insertion, participants in the treatment group will receive intrapleurally administered tissue plasminogen activator (tPA) 4 mg followed by dornase alfa 5 mg. Participants in the placebo group will receive tPA 4 mg followed by normal saline. Study treatments will be administered once daily for 3 days. All participants, parents or caregivers, clinicians, and research personnel will remain blinded. The primary outcome is length of stay from chest tube insertion to discharge from hospital. Secondary outcomes include time to meeting discharge criteria, chest tube duration, fever duration, need for additional procedures, adverse events, hospital readmission, cost of hospitalization, and mortality.

**Discussion:**

This multicenter randomized controlled trial will assess the safety, effectiveness, and cost-effectiveness of combined treatment with dornase alfa and fibrinolytics compared to fibrinolytics alone for the treatment of empyema in children.

**Trial registration:**

ClinicalTrials.gov: NCT01717742. Registered on 8 October 2012.

**Electronic supplementary material:**

The online version of this article (doi:10.1186/s13063-017-2026-0) contains supplementary material, which is available to authorized users.

## Background

Pneumonia is one of the most common reasons for children to be admitted to hospital and accounts for more inpatient costs than any other diagnosis outside of the newborn period [1, 2]. Up to 50% of these patients have an associated parapneumonic effusion [2]. Most are small and uncomplicated and will resolve with antimicrobial treatment of the underlying infection. In some cases, however, a complicated effusion can develop, leading to respiratory compromise and/or extensive loculations. Such effusions, commonly referred to as pleural empyema, lead to substantial morbidity, including respiratory distress, pain, and prolonged hospitalization, as well as child school loss, parental work loss, and stress on families. Over the past few decades, there has been a dramatic increase reported in the incidence of pediatric pleural empyema in multiple countries [3–11]. More recent data from Scotland suggests that since 2010, the incidence of empyema may have begun to fall, possibly due to the introduction of a 13-valent pneumococcal vaccine [12].

Published clinical practice guidelines support the use of pleural drainage procedures in addition to antibiotics for the management of moderate to large empyemas in children [13–16]. These guidelines are based on reports suggesting longer hospital stays, duration of antibiotic therapy, and higher rate of progression to surgical intervention in children treated with antibiotics alone [17]. A variety of drainage procedures have been described, including chest tube placement with or without fibrinolytics, repeated ultrasound-guided thoracentesis in addition to surgical procedures such as video-assisted thoracoscopic surgery (VATS), and open thoracotomy with decortication [13–16]. Systematic reviews and guidelines suggest that chest tube insertion with intrapleurally administered fibrinolytics and primary VATS are equally effective but fibrinolytics are more cost-effective [18, 19].

Even with the use of fibrinolytics, pleural drainage can be challenging and lead to prolonged hospitalization. Associated morbidities include prolonged chest tube duration, which can be painful, and “treatment failure,” where undrained pleural disease necessitates salvage procedures such as additional chest tubes, VATS, or thoracotomy. The frequency and type of salvage procedures varies by center but is typically 15% [20–22]. One potential explanation for inadequate drainage leading to treatment failure is the presence of extracellular uncoiled deoxyribonucleic acid (DNA) liberated from dead leukocytes and other bacterial components. Such residual material may increase viscosity, permit biofilm formation, and interfere with drainage [23–26]. Recombinant human dornase (dornase alfa) has been shown in vitro to decrease viscosity by cleaving free DNA and liquefying parapneumonic pus [26]. Subsequent animal studies have also demonstrated that the combined administration of the fibrinolytic tissue plasminogen activator (tPA) and dornase alfa is more effective than either agent alone [27]. Small case series in adult patients have also described benefit from the addition of dornase alfa to the treatment of empyema [28, 29]. Safety data on dornase alfa is primarily derived from its currently licensed indication, nebulization at a dose of 2.5 to 5 mg once or twice daily for the reduction of sputum viscosity in patients with cystic fibrosis. This formulation is generally well tolerated. Common side effects of inhaled dornase alfa include rash, voice alteration, chest pain, and laryngitis [30].

A randomized controlled trial of adults with empyema recently demonstrated improved outcomes with dornase alfa and fibrinolytics compared to fibrinolytics alone [31]. This study used a blinded, 2-by-2 factorial design in which 210 adults with empyema were randomly assigned to one of four study treatments for 3 days: double placebo, intrapleurally administered tPA and dornase alfa, tPA and placebo, or dornase and placebo. The primary outcome was the change in pleural opacity, measured as the percentage of the hemithorax occupied by effusion on chest radiography on day 7 compared with day 1. Secondary outcomes included referral for surgery, duration of hospital stay and adverse events. For the primary outcome, the authors found a significantly greater reduction in pleural opacity in the tPA-dornase alfa group compared with the placebo group (−29.5 ± 23.3% versus −17.2 ± 19.6%; mean difference −7.9%; 95% confidence interval (CI), −13.4 to −2.4; *p* = 0.005). The change in pleural opacity observed with tPA alone or dornase alfa alone was not statistically different from that observed with placebo. All secondary outcomes also pointed to superiority of tPA-dornase alfa compared with other study arms. Hospital stay for the tPA-dornase alfa group (11.8 ± 9.4 days) was about 50% shorter compared with placebo (24.8 ± 56.1 days), whereas in the dornase alfa-only group and the tPA-only group, length of stay was similar to placebo. The frequency of surgical referral at 3 months was lower in the tPA-dornase alfa group compared with placebo (4% versus 16%; odds ratio (OR), 0.17; 95% CI, 0.03 to 0.87, *p* = 0.03). Surgical referrals were increased in the dornase alfa-only group (OR, 3.56; 95% CI, 1.30–9.75, *p* = 0.01), and were nonsignificantly reduced in the tPA-only group (OR, 0.29; 95% CI, 0.07 to 1.29, *p* = 0.10). Mortality rates were 8% at 3 months and 12% at 12 months, but were similar across all study groups. Inflammatory measures (C-reactive protein, systemic white blood cell count, and odds of fever) were also assessed. Significant differences were found in mean white blood cell count on day 7 and fever on day 6 or day 7 between tPA-dornase alfa and placebo. The new treatment was not associated with any excess of adverse events. Serious adverse events described included intrapleural hemorrhage (*n* = 2, both in the tPA-dornase alfa group), gastrointestinal bleeding (*n* = 2, both in the dornase alfa group), hemoptysis (*n* = 1, in the tPA-dornase alfa group, and clinical deterioration (*n* = 1, in the placebo group).

Applying the results of adult studies of pleural empyema to children is problematic as pediatric pleural empyema is a different disease for several reasons. First, the mortality rates in adult patients can be as high as 10-20%, since many patients have pre-existing comorbidities [32, 33]. Most children who develop pleural empyema, however, are otherwise healthy and mortality is extremely rare. To date, no mortalities have been reported in any of the randomized controlled trials of children with empyema [20, 21, 33, 34]. Second, epidemiologic trends showing a rising incidence in empyema have been primarily demonstrated in children. Although microbiologic confirmation is often elusive, this does suggest that the microbial etiology of pediatric empyema (e.g., pneumococcal serotypes not covered by conventional vaccines) may differ from adult patients. Third, therapies that have been found to be ineffective in adult patients have been effective in children. Therefore, although there is a biological rationale and clinical efficacy data from adults, the evidence remains unclear whether the addition of dornase alfa to tPA will provide improvement in outcomes among children with empyema.

## Methods/design

### Research question

In previously well children who present with pleural empyema, does the administration of intrapleurally administered tPA and dornase alfa once daily for 3 days decrease the length of stay in hospital compared to tPA alone? We will also explore whether there are differences between treatment groups in terms of effectiveness, cost, and safety.

### Design

**Fig. 1:**
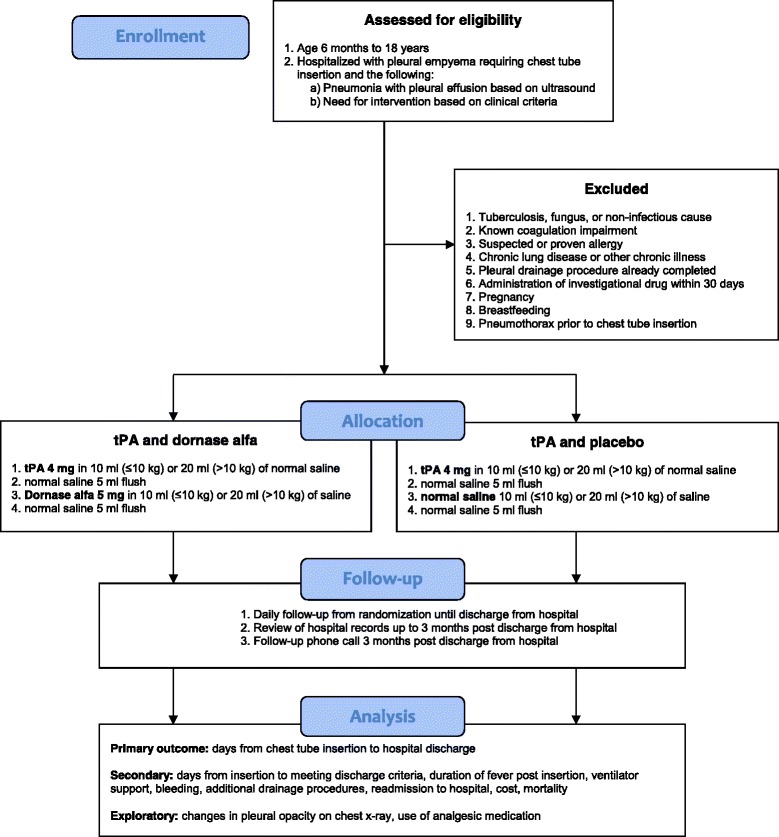
Overview of the intrapleural Dornase and Tissue Plasminogen Activator in pediatric empyema (DTPA) trial

**Fig. 2:**
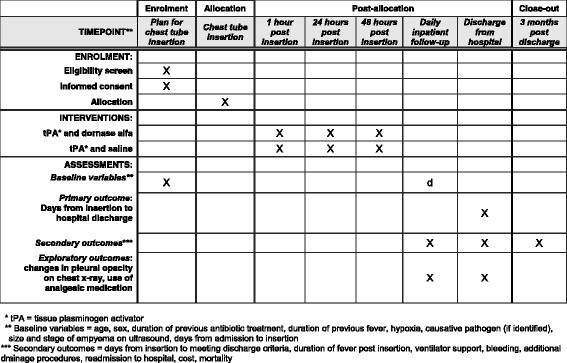
Overview of enrollment, interventions, and assessments as per the Standard Protocol Items Recommendations for Interventional Trials (SPIRIT) Statement [45]

The Intrapleural dornase alfa and Tissue Plasminogen Activator in pediatric empyema (DTPA) trial is a superiority, placebo-controlled, parallel-design, pragmatic, multicenter randomized controlled trial. This study will assess the safety, effectiveness, and cost-effectiveness of Dornase alfa combined with tPA compared to tPA alone. Overviews of enrollment, interventions, and assessments are depicted in Figs. 1 and 2 (which depicts the Standard Protocol Items Recommendations for Interventional Trials (SPIRIT) figure). The SPIRIT checklist is included in Additional file 1.

### Setting

The DTPA trial will be conducted at six tertiary children’s hospitals across Canada: The Hospital for Sick Children, Children’s Hospital of Eastern Ontario, Centre Hospitalier Universitaire Sainte-Justine, McMaster Children’s Hospital, British Columbia Children’s Hospital, and Alberta Children’s Hospital. These sites are the largest children’s hospitals in Canada and preferentially rely on chest tube insertion and fibrinolytics (as opposed to VATS) as first-line treatment. Furthermore, in all six centers, chest tube insertion is almost always performed using an image-guided, percutaneous technique by an interventional radiologist. Finally, all six centers have implemented clinical pathways to standardize the management of this condition.

### Participants

The DTPA trial will include children diagnosed with pleural empyema who require pleural drainage based on history, physical examination, laboratory investigations, and results of chest ultrasound. Current guidelines do not recommend that children with empyema undergo thoracentesis for the purpose of diagnosis prior to definitive pleural drainage via chest tube insertion or VATS [13–16].

### Inclusion criteria

1. Age 6 months to 18 years
2. Hospitalized with a diagnosis of pleural empyema requiring chest tube insertion and fibrinolytics (as judged by the attending physician) with the following criteria:
  - Pneumonia with pleural effusion based on chest ultrasound; and
  - Need for further intervention based on clinical criteria (persistent fever despite antibiotics for at least 48 h, significant respiratory distress, tachypnea, or hypoxia as a result of the pleural effusion)

### Exclusion criteria

1. Empyema as a result of tuberculosis, fungus, or noninfectious causes (e.g., malignancy)
2. Known coagulation impairment
3. Suspected or proven allergy to tPA or dornase alfa
4. Chronic lung disease or other chronic illnesses (e.g., immunodeficiency or neurologic impairment)
5. Child has already undergone a drainage procedure (e.g., chest tube or VATS)
6. Recent administration of an investigational drug (within previous 30 days)
7. Pregnancy
8. Breastfeeding
9. Presence of pneumothorax prior to chest tube insertion (i.e., possible bronchopleural fistula)

### Interventions

Participants will be randomized to receive either: (1) tPA (alteplase (Cathflo®), Roche) 4 mg followed by dornase alfa (Pulmozyme®, Roche) 5 mg or (2) tPA 4 mg followed by a placebo (saline). These medications will be administered through the chest tube once daily for a total of three doses. Weight-based dosing will not be used for tPA or dornase alfa as pleural concentrations are unpredictable and variable. Dornase alfa will be constituted by the research pharmacies in clear liquids in a polyethylene syringe. Since the stability of a tPA-dornase alfa admixture is unknown, the drugs will be administered sequentially with a 1-h indwelling time for each drug, as described in the trial of adults with empyema treated with dornase alfa and tPA [31].

For tPA, the contents of the vial will be diluted to a total volume of 10 ml normal saline for children weighing less than or equal to 10 kg and 20 ml for children weighing more than 10 kg. A flush of 5 ml of normal saline will be instilled after drug administration. Following the instillation of tPA, the chest tube will be clamped for 1 h and then opened to a negative pressure of −15 to −20 cm H_2_O for 1 h. Then, either dornase alfa or placebo will be instilled as a volume of 10 ml for children weighing less than or equal to 10 kg and 20 ml for children weighing more than 10 kg. These treatments will once again be followed by a 5-ml normal saline flush, 1 h of chest tube clamping, and then negative pressure to −20 cm H_2_O. In total, each administration of study drugs and drainage will take 4 h to complete.

The pharmacies will prepare the two arms (dornase alfa or placebo) in a manner such that both are identical (packaging, color, volume, texture, and odor) to ensure blinding. The contents of the vials (tPA followed by dornase alfa or placebo) will be instilled into the chest drain by clinicians caring for the child. The first dose of tPA will be given immediately (within 1 h) after insertion of the chest drain by the interventional radiologist or surgeon in the procedure suite or by clinicians on the ward. Dornase alfa or placebo will be instilled on the ward. On the following day (day 1) and subsequent day (day 2), a dose of tPA followed by the study drug will be administered in the morning, between 9:00 a.m. and 10:00 a.m., in a similar fashion. Thus, each patient will receive a total of three doses of either tPA or tPA-dornase alfa over 48 h or less. Participants will not receive any intrapleurally administered drug other than directed from this study.

### Criteria for discontinuing study interventions

Participants who develop anaphylaxis or serious hemothorax (requiring a transfusion or resulting in a hemoglobin drop of greater than or equal to 20 g/L) while receiving study interventions will not receive further study drugs. Participants whose chest tube is dislodged will only be replaced if clinically indicated for pleural fluid drainage and not for the sole purpose of study drug administration.

### Standard care

Participants will otherwise receive standard care, including supportive care, laboratory investigations, imaging, antibiotics, chest tube care and removal, and discharge. Although some variability is expected, rigorous randomization and blinding should ensure that confounders are equally distributed between groups. A detailed care map will be adapted from the existing clinical practice guideline co-authored by team investigators and other members of the Canadian Pediatric Society [35]. Specific elements in the care plan include:

- *Antimicrobial management*
  Participants in both treatment arms will receive standard antibiotic therapy for pleural empyema in children as directed by the most responsible physician. All participating hospitals currently recommend a second- or third-generation cephalosporin (cefuroxime, ceftriaxone, or cefotaxime) as empiric antimicrobial therapy with possible addition of cloxacillin, clindamycin, or vancomycin if *Staphylococcus aureus* is suspected. It is expected that antibiotic regimens may be tailored based on local microbiology patterns and sensitivities.
- *Chest tube insertion*
  Chest tubes will be placed by an interventional radiologist using an image-guided percutaneous technique or by another physician. The recommended size is an 8 or 10 French pigtail catheter.
- *Chest tube management*
  Management of chest tubes be dictated by the treating physicians. Suggested management includes maintaining −20 cm H_2_O of continuous suction and once daily flushes with 10 ml normal saline (on days when no study drug is administered) to maintain patency.
- *Diagnostic imaging post chest tube insertion*
  There is no consensus on the type (ultrasound versus plain radiograph) or frequency of diagnostic imaging required after chest tube insertion in hospitalized children. As such, decisions about follow-up imaging will be at the discretion of the responsible physician. Furthermore, results from follow-up radiographs will only be assessed as an exploratory outcome.
- *Criteria for chest tube removal*
  Our study protocol recommends that chest tubes be removed once drainage decreases to less than 1 mL/kg/day. Ultimately, however, the decision will be at the discretion of the responsible physician and likely based on a gestalt assessment of clinical parameters (e.g., amount of drainage, appearance of the child, fever, etc.).

### Primary outcome

The primary outcome will be length of stay from the time of chest tube insertion to discharge from hospital. This variable is the most common outcome reported in trials of empyema in children [20, 21, 33, 34]. In the current study, length of stay will be reported for each participant as days rounded to a single decimal point. All calculations will be based on the number of hours in hospital.

### Secondary outcomes

Secondary outcomes include measures of effectiveness, harm, and cost-effectiveness. This includes time to meeting discharge criteria, defined as the number of days from insertion of the chest drain to meeting discharge criteria. This will be assessed by a research assistant on a twice-daily basis (9:00 a.m. and 4:00 p.m.) and defined as: no fever (temperature <38 °C) for 24 h, normal respiratory rate for age (using the World Health Organization age-specific criteria: <50 breaths/min for 2–12 months, <40 breaths/min for 1 to 5 years, and <20 breaths/min for ≥5 years), no hypoxia (oxygen saturation greater than 92% on room air), and drinking fluids well. All values will be measured in hours and reported in days rounded to a single decimal point. We will use a similar approach to calculate days from chest tube insertion to removal.

We will also report the duration of fever after chest tube insertion. This will be defined as the length of time measured in hours and reported in days rounded to a single decimal point from chest tube insertion to the last recorded incidence of temperature >38 °C (recorded by any method). Dichotomous outcomes include in-hospital mortality due to any cause; need for ventilatory support or noninvasive ventilation following chest tube insertion; clinically significant bleeding (defined as intrapleural bleeding resulting in a drop in hemoglobin of greater than 20 g/L or requiring a transfusion of packed red blood cells); and the need for further pleural drainage procedures, including additional chest tube insertion, VATS, or thoracotomy. We will also document hospital readmissions within 3 months related to pleural empyema or its treatment.

Previous studies have demonstrated that early pleural drainage (i.e., within 48 h of admission) is associated with decreased length of stay in hospital [36, 37]. This finding has important implications regarding the timing of pleural drainage but does not help clinicians to determine which medications would be most beneficial (i.e., dornase and fibrinolytics versus fibrinolytics alone). As a result, we plan to report the timing of pleural drainage as a baseline variable to explore whether this is balanced between the two groups.

An economic evaluation will compare the relative costs of dornase alfa and tPA with tPA alone using participant-level data from the trial. We will conduct the analysis from a hospital’s perspective because hospital administrators will be making reimbursement decisions for this intervention. Since empyema is an acute condition, and the long-term consequences are negligible, the time horizon of the analysis will be the length of hospital stay. The cost for each patient includes the cost of intervention (dornase alfa and tPA or tPA alone) and costs incurred during the hospital stay (i.e., “hospitalization cost”). We will obtain hospitalization costs for each patient from case costing data from hospital finance departments. We will calculate mean cost per patient in each treatment group, based on initial intervention assignment, and incremental cost using simple linear regression:

$$ {C}_i = {\alpha}_i + \beta {t}_i+{\varepsilon}_i, $$

where *C*_i_ is the cost for each patient i, *α* is the intercept term, *t* is an intervention dummy term (*t* = 1 if the patient received dornase alfa-tPA and *t* = 0 for tPA alone), and *ε* is the stochastic error term. The regression coefficient *β* estimates the incremental cost of dornase alfa-tPA compared with tPA alone. The regression statistics will show mean cost per patient by intervention group and the uncertainty around the mean estimates. We will also conduct sensitivity analysis to assess the robustness of the results.

We will not subject participants to standardized diagnostic assessments in follow-up. The long-term outcomes of pediatric empyema are almost universally positive. In our previous prospective cohort study of children with empyema, we found that by 6 months virtually all patients were asymptomatic, radiographs had normalized in 95% of patients, and pulmonary function tests were normal in 96% [38].

### Exploratory outcome

In the trial of dornase alfa among adults with empyema, changes in pleural opacity, measured as the percentage of the hemithorax occupied by effusion on day 7 compared with day 1, was the primary outcome. In pediatric empyema, this outcome is problematic because: (1) some children will be discharged prior to day 7, (2) hemithorax size differs substantially across different-sized children, and (3) requiring an additional chest radiograph may be a substantial disincentive for a parental caregiver to provide consent given the rising concerns of ionizing radiation in developing children. Further, radiographic changes are considered surrogate measures of clinical changes as they often lag behind clinical improvement.

Chest ultrasound is not used routinely or consistently to assess response to therapy following pleural drainage procedures. Thus, using this modality in a pragmatic trial has little relevance for clinicians who routinely care for children with empyema. Further, ultrasound is extremely user-dependent, resource-intensive, and precludes truly blinded assessment by external reviewers. The most objective and reproducible way to assess response to pleural drainage radiologically would be chest computed tomography (CT). Unfortunately, chest CT is associated with less interobserver agreement for pleural disease, substantial cost, and radiation exposure, and so its use is not recommended in the management of children with pleural empyema [39].

There is currently no standard as to the timing of chest radiographs in hospital, but in our experience, virtually all children will have a radiograph performed before and/or shortly after chest tube removal. The radiograph closest to the time of removal will be reviewed by a blinded study radiologist to determine the percentage of hemithorax occupied using a five-point ordinal scale utilized in previous studies ranging from no fluid present to fluid occupying more than 75% of the most affected hemithorax. Given that radiographic improvement is time-dependent, we do not have any a priori hypotheses about the results of this outcome across groups. Using a chest radiograph allows for a blinded assessment by an external reviewer and is the modality used in routine clinical practice, particularly to ensure that the patient does not develop a pneumothorax and/or that the chest tube does not become dislodged or kinked while in situ. Thus, the degree of resolution on chest radiograph post chest tube insertion is both meaningful to clinicians and more in keeping with the goals of a pragmatic trial.

We are also collecting data on concomitant medication use, including analgesia. We did not include pain as a prespecified outcome measure as we did not hypothesize differences between treatment groups. We will explore these data in post hoc analyses.

### Sample size

The primary outcome in this trial is time to hospital discharge after chest tube insertion. In previous studies, the mean time to hospital discharge post insertion ranges from 6 to 15 days [20, 21, 33, 34]. Hospital length of stay tends to be shorter in published randomized controlled trials compared to observational studies of empyema, and a recent randomized controlled trial using an identical tPA dosing regimen to that which we are proposing described a mean of 6.8 (standard deviation (SD) 2.9) days [21]. The desired power for the current proposal is 90% to detect a difference of 2 days in the mean time to hospital discharge between the treatment arms. Based on discussions with clinical experts, hospital administrators, and parents of children with pleural empyema, it is believed that a 2-day difference between treatment groups is a minimal clinically meaningful difference, and this has been used in previous trials on this topic [38]. The adult dornase alfa trial found a 50% difference in length of stay between treatment and placebo groups [40].

Assuming a type 1 error rate of 0.05 (two-sided), power (1 − *β*) of 90%, and a SD of 2.9 days for each group, our trial needs 46 participants in each group to detect a difference of 2 days in length of stay. Sample size would have to be 34 participants in each group to provide 80% power. Calculations were performed based on a *t* test of independent groups. There will be no other adjustment to the sample size requirements due to loss to follow-up for the primary outcome as the primary outcome is assessed in hospital where the research coordinator will be able to ensure complete data collection.

### Recruitment

All study participants will be initially identified by research assistants who will review new admissions to the relevant inpatient units twice daily. They will also review new referrals for chest tube insertion at each center. Most chest tubes will be inserted by interventional radiologists, although a minority may be inserted by general surgeons or intensivists. Physicians, radiologists, and surgeons at each site will be asked to notify the research assistant and/or site investigator of children admitted with pleural empyema who may be eligible for the study. Potential participants will be approached, eligibility criteria confirmed, consent obtained, and enrolled after the decision to proceed with pleural drainage but before chest tube insertion occurs.

### Randomization

After informed consent is obtained, participants will be randomized into treatment groups using a random allocation sequence created by the coordination and management center for this trial. Randomization will be stratified by center. Blocking will be used to ensure that the two comparison groups are approximately the same size throughout the trial for each center and for the trial as a whole. An allocation ratio of 1:1 with random block size will be used within each stratum (i.e., center). This will ensure that clinicians, investigators, and outcome assessors will not be able to decipher the block size. A computer-based pseudo-random number generator will be used to create treatment allocation tables for each study center.

After patient eligibility has been confirmed, consent obtained, and just as the patient leaves the inpatient unit to the radiology suite or operating room for chest tube insertion, the site investigator (or delegate) will assign the patient a unique study identification number in sequential order. The study identification number will correspond with the randomization table held in the research pharmacy for dispensing blinded dornase alfa or placebo. The biostatistician will maintain a secure master list of the randomization codes and the assigned treatments will be checked against the master list at the end of the study.

### Blinding

Participants, parents or other caregivers, site investigators, research assistants and coordinators, treating physicians (pediatricians, radiologists, and interventional radiologists or surgeons), treating nurses, and data managers will be blinded to the treatment allocation. Group allocation will be concealed until the final data analysis is performed. Study drugs will be blinded by the research pharmacy. Both arms will be constituted by the research pharmacies in clear liquids in a polyethylene container in a manner such that both are identical (packaging, color, volume, texture, and odor) to ensure blinding. After obtaining the treatment number from the central randomization center, the study pharmacist will retrieve the corresponding vial and one of its treatment number labels will be attached to the participant’s Case Report Form. We do not anticipate any circumstances that would require unblinding as knowledge of the study arm is not anticipated to affect treatment for patients.

### Data collection

Data collection for outcome measures will be collected in hospital by the research assistant at each center. At baseline, the following data will be obtained for descriptive purposes: age, sex, duration of antibiotic treatment prior to chest tube insertion (in days), duration of fever prior to chest tube insertion, hypoxia while breathing room air (oxygen saturations less than 92% prior to intervention), microbiologic identification of causative agent (culture or polymerase chain reaction results from blood, throat swab, or pleural fluid), size of empyema on ultrasound, stage of empyema on ultrasound (stage 1: anechoic, nonseptated fluid; stage 2: echoic fluid without septation; stage 3: septated fluid; and stage 4: septations with solid appearing components comprising more than one third of the effusion). All ultrasounds will be reviewed by a blinded study radiologist. In addition, all participants will receive a follow-up phone call from the research assistant at 3 months enquiring about any possible readmissions and ongoing symptoms of fever, shortness of breath, and/or exercise intolerance.

### Data management

The Applied Health Research Center of the Li Ka Shing Knowledge Institute of St. Michael’s Hospital will serve as the data management center. The Applied Health Research Center employs state-of-the-art web-based data management software, Medidata RAVE™ (5.6.3), a secure, encrypted web-based clinical trial data management system which is fully configurable and incorporates sophisticated data validation rules to ensure high-quality data capture. RAVE™ allows for remote web-based data entry directly from the hospital sites, facilitating real-time data access.

### Statistical analysis

#### Baseline characteristics

Patient characteristics and descriptive variables will be presented for each treatment arm: age, sex, duration of antibiotic treatment prior to chest tube insertion (days), duration of fever prior to chest tube insertion (days), hypoxia (defined as the presence of saturation less than 92% without supplemental oxygen), bacterial identification and subtype, pleural effusion size on ultrasound (>10 mm or <10 mm), stage of empyema on ultrasound (frequency). For continuous variables, means and SDs or medians (interquartile ranges) will be presented. For categorical variables, proportions will be presented.

#### Primary outcome

Data will be analyzed according to intention-to-treat principles for the primary outcome (i.e., patients who do not receive all three doses of study drug will be analyzed in the group they were assigned to). Exceptions to this principle will only include any patient who dies in hospital and will be excluded from the analysis of time to hospital discharge. Given that the primary outcome and other acute secondary outcomes are obtained during hospitalization, it is anticipated that there will be no missing data for these outcomes with the possible, but unlikely, exception of in-hospital death which is rare in childhood empyema.

For the follow-up outcomes at 3 months, the proportion of patients with follow-up will be presented for each treatment arm, and patients who are lost to follow-up will be stated but omitted from the analysis. The primary outcome, time (in days) to hospital discharge, will be described as the difference between the two means with the 95% CIs. The Student’s *t* test (independent two-sample test assuming equal group size and variance), will be used to detect a difference between the two treatment groups. If the resulting data is inappropriate for a *t* test (increasing variance with the mean is the biggest concern since the test is quite robust to non-normality in the population), a suitable transformation or nonparametric test may be used. If feasible, a secondary analysis of the primary outcome will analyze time to discharge, treating death as a competing risk, using methods for survival data. Subgroup analyses will also be conducted to explore any potential differences in outcomes by age or sex.

#### Secondary and exploratory outcomes

For the secondary and exploratory outcomes that are continuous variables (i.e., duration of tube insertion, hospital stay after intervention to meeting discharge criteria, duration of fever after the intervention, amount of hemithorax occupied by pleural effusion in radiographs) the difference between the two means with the 95% confidence intervals will be presented. The Student’s *t* test will be used to detect a difference between the treatment groups. Dichotomous outcomes (serious bleeding, need for further interventions, need for ventilator support, mortality) will be described as the absolute number and proportion. A Fisher’s exact test will be used to detect a significant (*p* < 0.05) difference between the two treatment arms. The treatment effect will also be presented as the relative risk with 95% CIs. These analyses will be viewed as hypothesis-generating, and therefore, no correction for multiple testing is planned.

### Data monitoring and safety

Data monitoring will be conducted by a Data Monitoring Committee (DMC) composed of a pediatric hospitalist, a respiratory physician, and an interventional radiologist. The DMC will be completely independent of the investigators, and will be provided with clinical information from the Case Report Forms for death, surgical interventions, and adverse events, including those that were described in the adult dornase alfa trial (hemoptysis, gastrointestinal bleeding, chest pain, nausea, transient confusion, and rash). The DMC will be able to request additional information from clinicians as needed. All adverse events will be reported to the principal investigator within 24 h and to the local Research Ethics Board. Serious adverse drug reactions to the study medication will be reported to Health Canada within fifteen calendar days and within seven calendar days for life-threatening events or death. Adverse reactions will be managed by the treating physician and clinical team.

This project will be monitored by Applied Health Research Center during the data collection phase of the project. The aim is to ensure that all researchers are maintaining the highest ethical, scientific, and safety standards for all study participants, and are in compliance with all relevant policies, provincial and federal legislation, and international guidelines (such as Good Clinical Practice).

### Ethical considerations

Informed consent will be obtained from parents or legal guardians. Given the young age of children with empyema (average age less than 5 years), it is anticipated that most will not be able to consent or even assent to participate. Informed consent and assent will be obtained from all children who are able to provide it. Potential known adverse events from study interventions are mainly local as the drugs are not systemically absorbed. Aside from receiving the study interventions, participation will require parents to agree to be contacted by research personnel for a short phone call (duration less than 5 min) 3 months post discharge from hospital. Ethics review and approval will be obtained from the Research Ethics Board at each of the participating institutions.

### Dissemination

Knowledge Translation activities will occur locally, nationally, and internationally. Locally, findings will be presented to clinical groups and incorporated into the empyema Clinical Practice Guidelines at all sites. Nationally, findings will be presented at the Canadian Pediatric Society’s Annual Meeting focused on the Hospital Pediatrics Section. Internationally, we will present findings at the Pediatric Academic Society’s Annual Meeting, the largest international pediatric research meeting, facilitated by the Pediatric Research in Inpatient Settings group, an international hospitalist research and Knowledge Translation organization that is co-led by Dr. Mahant. Given the broad interest in empyema in children, we also plan to submit the results of our trial for publication in a high-impact general medical or pediatric journal.

## Discussion

There are important gaps in the scientific literature regarding the optimal therapy for pleural empyema in children [13, 14, 16]. This problem is pervasive in child health research. High-quality evidence is based on the results of randomized controlled trials but there is a paucity of these studies in children. Over the past 20 years, the number of adult randomized controlled trials published in leading general and subspecialty medical journals has increased substantially, while the number of pediatric trials has increased only modestly [41, 42]. This problem is particularly relevant in pharmaceutical trials, and has undoubtedly led to the widespread use of pharmaceutical products in children without sufficient data on effectiveness or safety [43]. Despite some legislative initiatives in the United States and Europe to promote drug trials in children, extensive logistical and financial disincentives persist. Consequently, 79% of hospitalized children are treated with drugs for unapproved indications, otherwise known as “off-label” use [44].

Randomized controlled trials have demonstrated that using fibrinolytics is a safe and effective therapy for children with empyema [20, 21, 34, 35]. Despite this, many patients continue to experience significant short-term morbidity due to prolonged hospitalization, pain from indwelling chest tubes, and the need for additional drainage procedures. Data from a randomized controlled trial in adults with empyema suggests that adding dornase alfa may be superior to using fibrinolytics alone. This treatment strategy requires further study in a pediatric setting since differences in the underlying disease may affect response to treatment. As such, we are now conducting a multicenter randomized controlled trial to assess the safety, effectiveness, and cost-effectiveness of dornase alfa and tPA in the management of empyema in children.

## Trial status

The DTPA trial started recruiting participants in December 2012 and is on track to complete enrollment of 92 participants by April 2018. An update with results will be provided in late 2018. A copy of the full-length protocol is available upon request.

## Additional file

Additional file 1: Recommended items to address in a clinical trial protocol and related documents. (DOCX 47 kb)

## Abbreviations

CI: Confidence interval
DMC: Data Monitoring Committee
DTPA: Intrapleural Dornase and Tissue Plasminogen Activator in pediatric empyema
OR: Odds ratio
tPA: Tissue plasminogen activator
VATS: Video-assisted thoracoscopic surgery
